# Identification of Serum CMTM2 as a Potential Biomarker for HBV-Related Disorders

**DOI:** 10.1155/2020/2032056

**Published:** 2020-10-08

**Authors:** Shu Chen, Qin Hu, Hong Chen, Fei-Fei Zhang, Liang Duan, Bo Wang, Dan-Dan Li, Juan Zhang, Wei-Xian Chen

**Affiliations:** ^1^Department of Laboratory Medicine, The Second Affiliated Hospital of Chongqing Medical University, Chongqing 400010, China; ^2^Department of Laboratory Medicine, Chongqing Traditional Chinese Medicine Hospital, Chongqing 400021, China

## Abstract

Substantial advance supports that CMTM2 serve as an important performer in physiological and pathological processes. However, very little is clear about the relationship between CMTM2 and HBV-related disorders. Here, for the first time, we explore that whether or not serum CMTM2 is involved in HBV-related diseases. We found that CMTM2 values were significantly lower in patients compared to healthy control (p <0.001), using ELISA assay. Furthermore, serum CMTM2 levels were negatively correlated with HBV DNA levels in CHB patients but not correlated with the serum levels of ALT and AST. Serum CMTM2 concentrations were not correlated with the serum levels of ALT, AST and HBV DNA load in HBLC and HCC patients. In addition, analysis of the ROC curve indicated that CMTM2 levels were significantly associated with the diagnostic value of HBV-related disorders. Finally, downregulation of CMTM2 was observed in HBV-infected cell model. CMTM2 degradation could be attributed to HBx-activated Lys48 (K48)-linked polyubiquitination, which was abolished by treatment with the proteasome inhibitor MG132. HBV infection suppresses CMTM2 expression by activating ubiquitin-proteasome system. Serum CMTM2 levels can be adopted as an effective indicator of the pathogenesis of HBV-related disorders.

## 1. Introduction

Hepatitis B virus (HBV) infection has been a growing public health problem of global attention at present. Approximately 250 million people worldwide are chronically infected with HBV [1]. Long-term HBV infection leads to acute and chronic hepatitis and even develops HBV complication, such as liver cirrhosis and hepatocellular carcinoma (HCC). However, the concrete mechanisms by which the pathogenesis of chronic HBV infection remain unclear.

CKLF-like MARVEL transmembrane domain-containing family (CMTM) is a novel protein family linking chemokines and members of the transmembrane 4 superfamily (TM4SF) [2]. In human, nine members, CKLF and CMTM 1-8 have important functions across a wide range of different physiological and pathological processes [3]. In recent years, with in-depth study, CMTM family may be of significant clinical value in immune system, male reproductive system and hematopoiesis system and even be involved in multiple malignancies [4].

As one of this family, CMTM2 is highly expressed in testis, bone marrow, and peripheral blood cells [5]. It has reported that CMTM2 participated in a wide range of biological processes, including hematopoiesis [5], spermatogenesis [6] and membrane apposition [3]. CMTM2 might be a potential antioncogene in embryonal carcinoma, yolk sac tumor [7] and non-small cell lung cancer [8]. Importantly, the role of CMTM2 in virus has also received much attention. For example, CMTM2 was reported to negatively control HIV-1 transcription by targeting the AP-1 and CREB pathways in T-cells [9]. However, it is noteworthy that very little is known about the role of CMTM2 in HBV-related disorders.

In this report, we aim to explore the changes of serum CMTM2 in patients with HBV-related liver diseases and get a better insight into the association between serum levels of CMTM2 and HBV-related complications. Meanwhile, depressed levels of CMTM2 in HBV-infected cell model and the precise mechanism were determined.

## 2. Materials and Methods

### 2.1. Patients and Sample Collection

From June 2018 to May 2019, 69 chronic hepatitis B (CHB) patients, 77 hepatitis B associated liver cirrhosis (HBLC) patients, 53 HCC patients and 52 healthy subjects (HCs) were enrolled in this study. The diagnosis of CHB was based on detectable hepatitis B surface antigen (HBsAg)-positive or HBV-DNA for more than six months. The diagnosis of cirrhosis and HCC was confirmed by histological examination of the resected material from patients. Written informed consent was obtained from each subject. The study was approved by the institutional ethics Committee for human studies at the Second hospital affiliated to Chongqing Medical University. Clinicopathological variables of the subjects at initial diagnosis in this study, including age, gender, HBsAg, CMTM2 values, ALT, AST, and HBV DNA load were collected. Serum HBsAg levels were measured by a chemiluminescence microparticle immuno assay by HBsAg reagent kit (Abbott, Chicago, USA). HBV DNA levels were detected using the LightCycler 480 real-time PCR system (Roche, Basel, Switzerland). ALT and AST levels were measured by Hitachi Modular 7600 chemistry analyzer (Hitachi, Tokyo, Japan) with test kit (Maccura, Sichuan, China), which based on UV-monitoring method. The patient characteristics are shown in Table 1. Serum samples were collected from 1 ml of coagulated blood after centrifugation and were frozen at -80°C until analysis.

### 2.2. Cell Culture

The liver cancer line cells HepG2 and HepG2.2.15 were routinely grown in Dulbecco's modified Eagle's medium (Hyclone, USA) containing 10% FBS (Lonsera, Uruguay) supplemented with 1% penicillin/streptomycin. All cells were grown at 37°C in a humidified incubator containing 5% CO_2_.

### 2.3. Plasmids and Transient Transfections

The expression vectors pcDNA3.1-HBV (1.3 or 1.1), pcDNA3.1, HBp, HBc, HBs and HBx were constructed in our laboratory. The overexpressed plasmid pET-T4090-M02-CMTM2 and control vector pET-T4090-M02-Ctrl were purchased from Labcell biotechnology (Chongqing, China). Cells grew to 70% to 90% confluence before treatment. Transfection of HepG2 cells with target plasmid or its control plasmid was conducted using lipofectamine 2000 (Invitrogen) according to the manufacturer's recommendation. And the cells were collected after transfection for indicated time to carry out follow-up studies.

### 2.4. ELISA

CMTM2 levels in serum and culture supernatants were measured by ELISA kit (Mengbio, Wuhan, China) following the manufacturer's recommendation.

### 2.5. Western Blot

The cells were collected and washed with ice-cold PBS, then lysed on ice in lysis buffer supplemented with 1% protease inhibitor and 0.5% PMSF. The protein concentrations were determined using the BCA assays and then dissolved in SDS electrophoresis buffer so as to heat in water bath at 100°C until protein degradation. The follow steps were electrophoresis separation, membrane transfer, and skimmed milk blocking. After that, membranes were incubated 24 h at 4°C using primary antibodies, subsequently incubated with the corresponding secondary antibody conjugated with horseradish peroxidase. Immunoblots were detected with the ECL Western blot reagents kit (Millipore, Massachusetts, USA). The results were analyzed by the Bio-Rad Electrophoresis Documentation (Gel Doc 1000, Bio-Rad, USA) and Quantity One Version 45.0. The antibodies used in this study are listed as follows: Rabbit anti-CMTM2 polyclonal antibody from Immunoway Biotechnology (Delaware, CA), Mouse anti-HBx monoclonal from Santa Cruz Biotechnology (Santa Cruz, CA), anti-*β*-actin monoclonal antibody, Horseradish peroxidase-conjugated anti-mouse, and anti-rabbit IgG antibodies from Boster Biological Technology (California, USA).

### 2.6. Real-Time Quantitative PCR

The total DNA was isolated using the TRIzol reagent (Invitrogen, Carlsbad, CA, USA). Single-stranded template DNA was synthesized from total RNA by using PrimeScript RT reagent Kit (TaKaRa, Japan). The levels of mRNA were evaluated by real-time polymerase chain reaction (PCR). The PCR primers were as follows: CMTM2 primers: (upstream) 5'-cgtggtctttgctgtgagaa-3' and (downstream) 5'-gggtcctttttcctttcctg-3'; *β*-actin primers: (upstream) 5'-tccctggagaagagctacga-3' and (downstream) 5'- agcactgtgttggcgtacag-3'. Every sample was repeated 3 times using SYBR Green master mix (TaKaRa, Japan). The relative expression levels of CMTM2 gene was normalized to *β*-actin and calculated using the 2^-∆∆Ct^ method.

### 2.7. Luciferase Reporter Assay

The experimental objects were divided into 2 groups: A. HBx + pEZX-CMTM21-promotor+pRL-TK; B. pcDNA3.1 + pEZX-CMTM2-promotor+pRL-TK. The vector pRL-TK was served as the internal reference. The above plasmids in each group were used for transfection of HepG2 cells. After 48 h of transfection, cells were lysed and relative luciferase activity was measured with a Dual Luciferase Assay (Promega, USA) according to the manufacturer's protocol. Relative luciferase activity was calculated as: Firefly luciferase vs. Renilla activity.

### 2.8. Ubiquitination Assays

HepG2 cells transfected with HBx expression vector or empty vector pcDNA3.1 were incubated in the presence or absence of 30 *μ*M MG132 (Cell Signaling Technology, Danvers, Massachusetts, USA) for 24 h. After that, the cells were lysed in RIPA lysis buffer (Beyotime, China) with 1% phenylmethylsulfonyl fluoride and 5% N-ethylmaleimide. A 60 mg aliquot of protein was retained as the total protein. After that, three microliters of anti-CMTM2 antibody was added to total cell lysate and incubated overnight with rotation at 4°C. Next, protein A/G was added and the mixture was incubated for 5 h at 4°C. The total protein and mixture were used for Western blotting. Ubiquitin antibody was obtained from Santa Cruz Biotechnology (Santa Cruz, CA). K48-linked ubiquitin and K63-linked ubiquitin antibodies were from Millipore (Billerica, MA, USA).

### 2.9. CCK8

Cell proliferation was evaluated using the cell counting kit-8 (Dojindo Laboratories, Kumamoto, Japan) assay according to the manufacturer's instructions. Briefly, 2000 cells transfected with CMTM2 plasmid or SiRNAs were seeded into 96-well plates and cultured for the indicated time. Then 10 *μ*L of CCK-8 solution was added into each well and incubated for 2 h. The absorbance at 450 nm was measured to assess the number of viable cells. The results were obtained from three independent experiments in triplicate.

### 2.10. Flow Cytometry

Cell apoptosis was evaluated using Annexin V-PI staining. Cells receiving different treatment were harvested, washed twice with ice-cold PBS and stained with Annexin V-FITC and propidium iodide according to the manufacturer's protocol. Cell apoptosis was measured by BD FACS Canto™ II flow cytometer. For the cell cycle analysis, the cells were harvested 48 h after transfection with the CMTM2 plamid or SiRNAs. Cell cycle was detected by propidium iodide (PI) staining followed by flow cytometry analysis, as previously described [10] .

### 2.11. Transwell Migration and Invasion Assays

Cell migration and invasion were assayed using Transwell chambers. Cells in 200 *μ*L DMEM containing with 1% FBS were added to the upper chamber, 500 *μ*L of DMEM containing 10% FBS was added in the lower chamber. After a 24 h incubation at 37°C in a 5% CO_2_ humidified atmosphere, the nonmigrated cells were scraped off of the filter using a cotton swab and the migrated cells were stained with crystal violet following fixation with 4% paraformaldehyde. In addition, for invasion assay, the chamber was pre-coated with Matrigel. The cells were quantified by counting 5 randomly fields of each well.

### 2.12. Statistical Analysis

All statistical analyses were conducted using GraphPad Prism software (GraphPad Software, CA, USA) and SPSS program (version 17.0). The clinical characteristics of human subjects were presented as means±SD. The data between two groups were compared by the Student's t-test. Receiver operating characteristic (ROC) curves were plotted to investigate the diagnostic role of serum CMTM2 for HBV-related disorders via calculation of the area under the ROC curve (AUC). Statistical differences were showed to be at different levels of p <0.05 (∗) and p <0.001 (∗∗∗).

## 3. Result

### 3.1. Serum Levels of CMTM2 in Patients and Healthy Individuals

**Figure 1:**
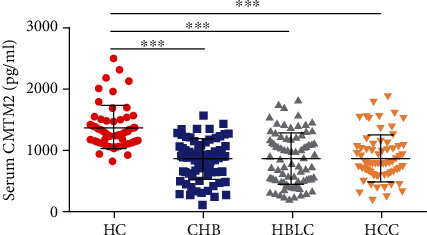
Serum CMTM2 concentrations in patients with HBV-related liver diseases. ELISA analysis of CMTM2 levels from serum samples of patients with HBV-related liver diseases at different phases (CHB, HBLC and HCC) as well as healthy controls (HCs). Data represents the mean ± SD, ^∗^p <0.05; ^∗∗∗^p <0.001.

To identify whether serum CMTM2 could be abnormally altered in patients with HBV-related liver diseases, we analyzed CMTM2 levels in four groups, including CHB, HBLC, HCC and healthy individuals. As shown in Figure 1, the serum concentration of CMTM2 in patients with HBV-related liver diseases was significantly lower than those of healthy individuals.

### 3.2. Correlation between Serum Levels of CMTM2 and Viral Replication in HBV-Related Liver Diseases

**Figure 2:**
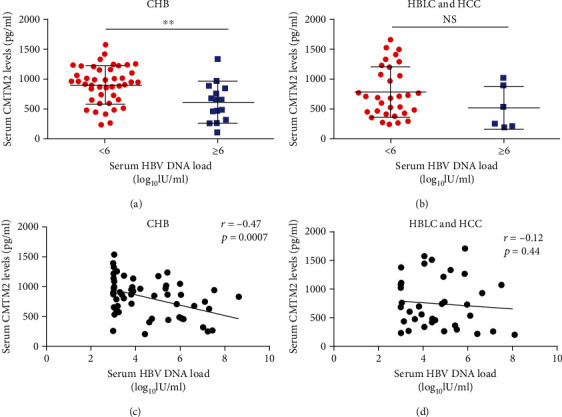
Correlation between serum levels of CMTM2 and HBV DNA load. Distribution of serum CMTM2 in CHB (a), HBLC and HCC (b) patients with low or high viral load (<6 and ≥6 log10 IU/ml). Correlation between serum CMTM2 and HBV DNA load in CHB (c), HBLC and HCC(d) patients. Data represents the mean ± SD, ^∗∗^p <0.01; NS: no statistical significance.

To gain better insight into the relationship between serum CMTM2 levels and HBV-related complications, we assessed whether or not CMTM2 is correlated with HBV viral load. According to HBV DNA levels, CHB patients was divided into two subgroups, including high virus load (≥6 log10 IU/ml) and low virus load (<6 log10 IU/ml). Average concentration of serum CMTM2 was proportionally changed to the increase of serum HBV viral load in CHB patients (Figure 2(a)). However, the serum levels of CMTM2 were not proportionally changed to the increase of serum HBV DNA in HBLC and HCC patients (Figure 2(b)). We next assessed the interplay of serum CMTM2 levels with viral loads. In these experiments, a moderately negative correlation was found between CMTM2 and HBV viral load in CHB patients (Figure 2(c)). Regarding patients with HBLC and HCC, there was no correlation between serum CMTM2 and HBV viral load (Figure 2(d)).

### 3.3. Correlation between Serum Levels of CMTM2 and Necroinflammation Parameters

**Figure 3:**
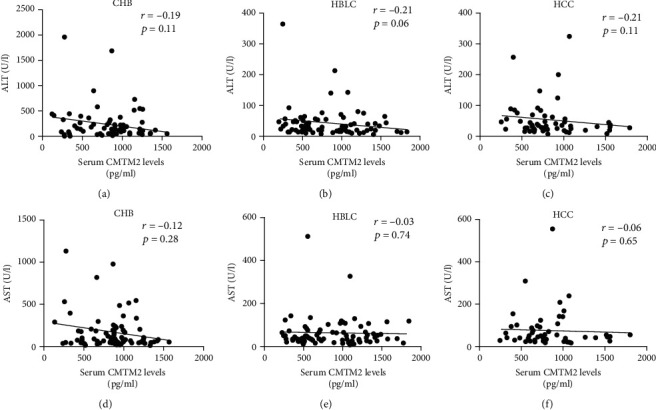
Correlation between serum levels of CMTM2 and necroinflammation parameters. Correlation between serum CMTM2 levels and ALT levels in CHB (a), HBLC (b), and HCC (c) patients. Correlation between serum CMTM2 levels and AST levels in CHB (d), HBLC (e), and HCC (f) patients.

Next, we further analyzed whether or not CMTM2 was correlated with necroinflammation parameters, including ALT and AST. In this work, the analysis indicated no significant association between serum CMTM2 and ALT in patients with CHB, HBLC and HCC (Figures 3(a)-3(c)). Additionally, a same phenomenon with regard to the correlation between serum CMTM2 and AST was found in patients with HBV-related complications (Figures 3(d)-3(f)).

### 3.4. Downregulated Expression of CMTM2 in HBV-Infected Cell Model

**Figure 4:**
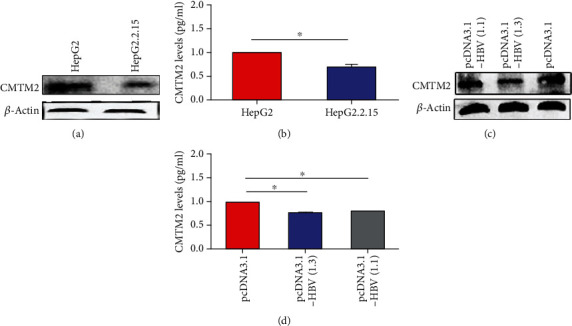
Decreased CMTM2 expression in HBV-infected hepatic cells. The protein levels and cultural supernatants levels of CMTM2 in HepG2 cells and HepG2.2.15 cells were determined by western blot and ELISA (a, b). *β*-Actin was used as an internal control in Western blot. After transfected with HBV expressing plasmid pcDNA3.1-HBV (1.1 and 1.3) or empty plasmid pcDNA3.1 into HepG2 cell, both western blot and ELISA assay were used to measure protein levels and cultural supernatants levels (c, d). ^∗^p <0.05; NS: no statistical significance.

The protein expression and cultural supernatants levels of CMTM2 were examined in HepG2 and HepG2.2.15 line cells (Figures 4(a)-4(b)). HepG2.2.15 is a HBV stably transfected cell line constitutively producing HBV. HBV 1.3-fold genome plasmid (pcDNA-HBV1.3) and HBV 1.1-fold genome plasmid (pcDNA-HBV1.1) were transfected into HepG2 cell line to establish a HBV-infected cell model (Figures 4(c)-4(d)). Both protein and cultural supernatants levels of CMTM2 were downregulated in HBV-expressing cells relative to control cells.

### 3.5. HBx Inhibited CMTM2 Expression

**Figure 5:**
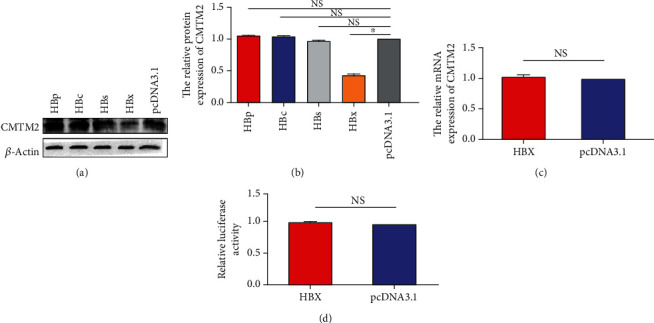
HBx inhibited CMTM2 expression. After transfected with four HBV viral protein plasmids (HBp, HBc, HBs and HBx) or empty plasmid pcDNA3.1 into HepG2 cell, respectively, the protein levels of CMTM2 were detected using Western blot (a, b). After transfected with HBx plasmid or empty plasmid pcDNA3.1 into HepG2 cell, the mRNA level was determined by real-time PCR (c). Relative luciferase activities were measured according to standard procedures after that the CMTM2 promoter luciferase reporter and HBx or pcDNA3.1 plasmids were co-transfected into HepG2 cells (d). ^∗^p <0.05; NS: no statistical significance.

To verify how HBV suppress CMTM2 expression, plasmids encoding various HBV proteins including HBc, HBs, HBx and HBp were transfected into HepG2 cells, respectively, and the protein levels of CMTM2 were detected by western blot. We found that CMTM2 expression decreased drastically after transfection with the HBx-encoding plasmid (Figures 5(a)-5(b)). To determine whether HBx could affect CMTM2 transcription, PCR and luciferase reporter assay were performed after transfection. The results showed that no inhibitory effects of CMTM2 mRNA and luciferase activity were observed in this work (Figures 5(c)-5(d)). Taken together, the results demonstrated that HBx could decrease CMTM2 protein level whereas do not affect its transcription. HBx might regulate CMTM2 through influencing protein stability and degradation.

### 3.6. HBx Induced Ubiquitination-Mediated CMTM2 Degradation

**Figure 6:**
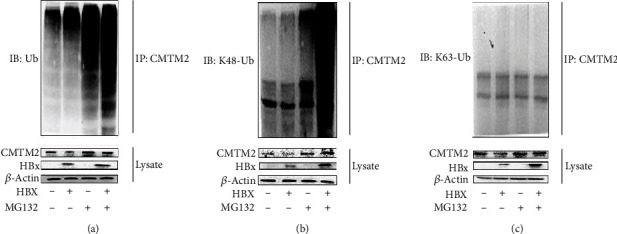
HBx induced ubiquitination-mediated CMTM2 degradation. HBx enhanced CMTM2 ubiquitination in HepG2 cell (a). K48-linked CMTM2 ubiquitination and K63-linked CMTM2 ubiquitination were immunoprecipitated with CMTM2 antibodies and detected by immunoblotting (b, c).

Ubiquitin-proteasome system was reported to be one of the major intracellular pathways for protein degradation, we then studied whether HBx affect CMTM2 by ubiquitin-proteasome pathway. We detected and analyzed the immunoprecipitates and ubiquitylation of CMTM2 in cell lysates of HBx-transfected HepG2 cells by western blot. MG132, a proteasome inhibitor, abolished the HBx-induced downregulation of CMTM2 protein in HepG2 cells. The levels of CMTM2 polyubiquitination in HepG2 cells was significantly increased after HBx transfection but the protein amount of CMTM2 was not affected by HBx when MG132 was supplemented (Figure 6(a)). Additionally, we also explored whether HBx induced CMTM2 degradation by the K48-linked polyubiquitination or K63-linked polyubiquitination pathway. The ubiquitination assays results indicated that HBx promoted K48-linked polyubiquitination of CMTM2 but not K63-linked polyubiquitination in HepG2 cells (Figures 6(b)-6(c)). Therefore, these results demonstrated that K48-linked polyubiquitination pathway is responsible for HBx-induced CMTM2 degradation.

### 3.7. In Vitro Analysis of Correlation between CMTM2 and Hepatocellular Carcinoma Progression

**Figure 7:**
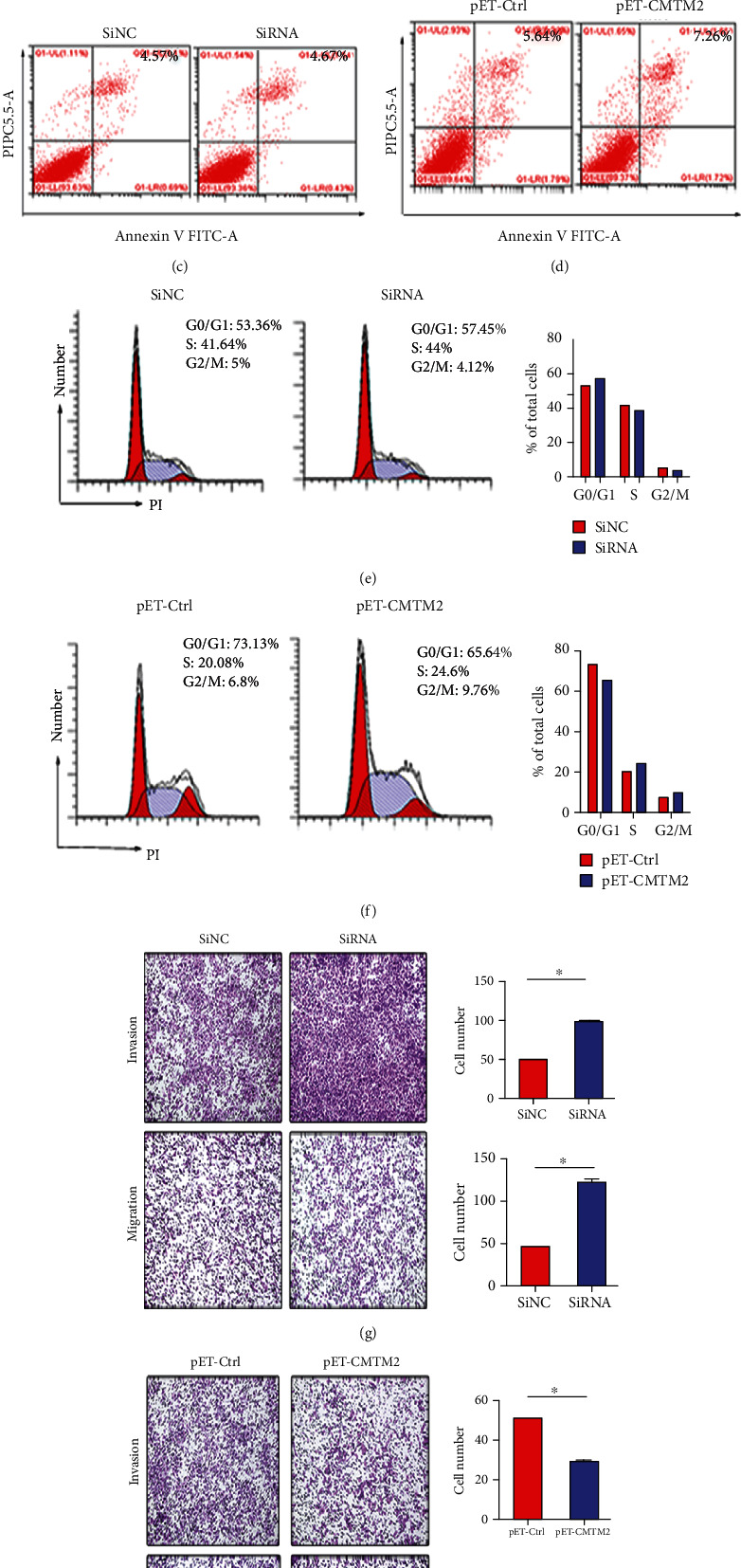
Correlation between CMTM2 and hepatocellular carcinoma progression. The Cell Counting Kit-8 assays showed that knockdown of CMTM2 promoted the cell growth, and cell growth was inhibited by overexpression of CMTM2 (a, b). Annexin V/PI-staining indicated that CMTM2 did not induce apoptosis of HepG2 cells (c, d). Knockdown of CMTM2 decreased the G2/M phase accumulation and overexpressed CMTM2 increased the G2/M phase population (e, f). Knockdown of CMTM2 could promote cell migration and invasion which were examined by Transwell migration and invasion assays (g). Overexpressed CMTM2 could inhibit cell migration and invasion of HepG2 cells which determined by Transwell migration and invasion assays (h). ^∗^p <0.05.

We next attempt to determine whether or not downregulation of CMTM2 expression correlated with HBV-related HCC. The CCK-8 assays showed that knockdown of CMTM2 with SiRNA in HepG2 cells was more potent than the negative control (SiNC) in promoting cell growth and overexpressed CMTM2 significantly inhibited the proliferation of HepG2 cells compared with control cells (Figures 7(a)-7(b)). To elucidate the molecular mechanisms underlying the tumour cell growth inhibition by CMTM2, its effects on apoptosis and cell cycle progression was analyzed using flow cytometry. Knockdown or overexpression of CMTM2 did not induce apoptosis of HepG2 cells. Knockdown of CMTM2 reduced the G2/M phase accumulation, and overexpression of CMTM2 increased the population in the G2/M phase compared with the control transfection (Figures 7(c)-7(f)). Taken together, these results revealed that CMTM2 cell cycle arrest at the G2/M phase without inducing cell apoptosis. Additionally, transwell migration and invasion assays were performed to explore the effect of CMTM2 on motility of liver cancer cells. SiRNA targeting CMTM2 brought obvious elevation of cell migration and invasion ability compared with SiNC group. Concordantly, the cell migration and invasion ability of CMTM2-transfected cells was remarkably reduced compared with that of control-transfected cells (Figures 7(g)-7(h)).

### 3.8. Differentiating Power of CMTM2 for Progression HBV-Related Disorders

**Figure 8:**
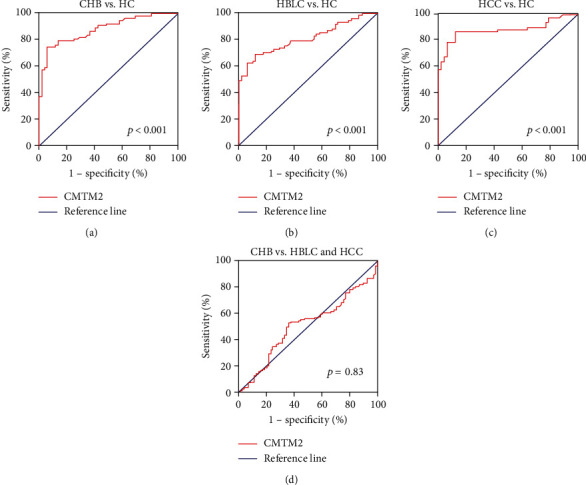
Differentiating power of CMTM2 for progression of HBV-related disorders. Receiver operating characteristic (ROC) curve of CMTM2 for detecting CHB patients from HCs (a). ROC curve of CMTM2 for detecting HBLC patients from HCs (b). ROC curve of CMTM2 for detecting HCC patients from HCs (c). ROC curve of CMTM2 for detecting HBLC and HCC patients from CHB (d).

Receiver-operating characteristic (ROC) curves were established, and the areas under the ROC curves (AUC-ROCs) were calculated to evaluate the discriminatory power of CMTM2 to distinguish CHB, HBLC and HCC from HCs. The ROC curve revealed that the area under the curve (AUC) value in the CHB and HCs was 0.88 (95% CI, 0.82 to 0.94), with 75.36% sensitivity and 95.23% specificity (Figure 8(a)). The same ROC analysis was performed to discriminate between HBLC patients and the normal group. The ROC curve showed that the AUC value in the HBLC and HCs was 0.81 (95% CI, 0.74 to 0.88), with 68.83% sensitivity and 88.46% specificity (Figure 8(b)). The AUC value in the HCC and HCs was 0.88 (95% CI, 0.81 to 0.95), with 86.79% sensitivity and 88.46% specificity (Figure 8(c)). The above data shows that CMTM2 performed a strong potential diagnosis value for differentiating the HBV-related disorders groups from the normal group. We also studied the predictive role of serum CMTM2 for HBLC and HCC from CHB. However, ROC analysis demonstrated an AUC of 0.50 (95% CI, 0.42 to 0.59), with 53.08% sensitivity and 63.77% specificity, which indicated that CMTM2 had no diagnostic value for identifying HBLC and HCC from CHB (Figure 8(d)).

## 4. Discussion

Over the past few decades, substantial evidence has been collected that lower expression of CMTM farmily was involved in a variety of cancers, including liver, lung, renal, gastric, ovarian, prostate, and oral squamous cell carcinomas [4]. For CMTM2, Mays AC et al. [11] found that CMTM2 may play important roles in the development of tumor metastases in Salivary Adenoid Cystic Carcinoma. The hypermethylation of CMTM2 promoter with concomitant transcriptional downregulation might perform tumor suppressive functions in Sézary syndrome [12]. Moreover, growing data indicates that CMTM2 might have unknown immune response. This assumption is mainly based on the research that CMTM2 exhibits the cell-chemotaxis on PC-3, U937, TM4 cell lines and murine splenic cells, together with CMTM2 expression in peripheral blood cells and bone marrow [13]. Recently, CMTM2 was discovered to play a pivotal role in some virus infection. For instance, CMTM2 may be biologically relevant in suppression of HIV-1 replication [9]. However, until now, whether or not CMTM2 is involved in HBV-related liver disorders remains to be elucidated. In this study, we explored the association of serum CMTM2 with viral replication and liver-specific enzymes, as well as the regulatory effect of HBV on CMTM2 expression in vitro.

Our results provide strong evidence that the serum CMTM2 values are significantly lower in patients with HBV-related disorders compared to healthy individuals. Furthermore, our findings show that the average concentration of serum CMTM2 was proportionally changed to the increase of serum HBV viral load and a moderately negative correlation between CMTM2 and HBV viral load was observed in CHB patients. However, the data indicates that average levels of serum CMTM2 was not proportional to the increase of serum HBV DNA and no correlation is present between CMTM2 and HBV viral load in HBLC and HCC patients. In such a case, we could observe the same phenomenon that average level of serum CMTM2 in high virus load samples is lower than that of in low virus load samples during HBLC and HCC phases. Since high load of HBV DNA is rarely detected in patients with HBLC and HCC, the sample sizes of high virus load in HBLC and HCC are small, which may cause difference with no significance. Currently, liver-specific enzymes such as ALT and AST are the most common serum biomarkers for hepatic function assessment [14]. Here, our results found no direct correlations between serum CMTM2 and ALT or AST in patients suffered from HBV-related disorders. Therefore, the serum CMTM2 levels of HBV-related liver disorders may be related to HBV replication, but not related to the degree of inflammatory injury.

As one type of post-translational modification, the main function of the ubiquitin-dependent pathway is mediating the degradation of cellular proteins.The ability of ubiquitin to form up to eight different polyubiquitin chain linkages generates complexity within the ubiquitin proteasome system [15]. K48-linked polyubiquitin chain is well established as the canonical signal for proteasomal degradation. So that, ubiquitin-modified proteins can be specifically recognized by the K48-linked polyubiquitin chain and subsequently degraded by the 26S proteasome in a classical pathway [15]. In this study, the data indicated that the viral protein HBx promote CMTM2 degradation by activating K48-linked polyubiquitination. Previous studies reported that HBx increase the ubiquitination of *β*-catenin via the activation of a p53-Siah-1 proteasome pathway in the presence of p53 and mediate the degradation of insulin receptor substrate 1 (IRS1) via ubiquitination to inhibit insulin signaling in hepatocytes [16, 17]. Additionally, it's reported that HBx targets SMC5/6 for ubiquitylation by the CRL4 *^HBX^* E3 ligase and subsequent degradation by the proteasome [18]. Here, we speculate that complicated interactions between the HBV and the host ubiquitin-proteasome system (UPS) may be responsible for CMTM2 degradation, but this observation stimulates the need to explore in more depth the mechanism underlying this.

Next, we show that negative expression of CMTM2 may participate in the progression of hepatocellular carcinoma. Several members, such as CMTM3, CMTM4, CMTM5 and CMTM7, have been documented to exhibit tumor suppressor functions in hepatocellular carcinoma cells [19–22]. In the present study, restoring CMTM2 expression not only inhibited liver cancer cell growth via G2/M phase accumulation without inducing apoptosis, but also suppressed cell metastasis and invasion in vitro.

The earlier study indicated CMTM2 had a secreted form [5]. Our results also reveal that CMTM2 can be detected in human serum. The relative expression of serum CMTM2 in patients with HBV-related disorders was significantly lower than healthy individuals. Here, ROC curve analysis showed that the AUC of using serum CMTM2 to distinguish between CHB and HCs was 0.88, which imply that serum CMTM2 could efficiently discriminate CHB patients from HCs. Similarly, the ROC plot yielded AUC of 0.81 and 0.88 between HBLC vs HCs and HCC vs HCs, respectively, indicating that the serum CMTM2 had a high value for identifying patients with HBLC and HCC from HCs, while it had no diagnostic value for identifying HBLC and HCC from CHB. So, our findings suggest that CMTM2 might be a new biomarker candidate for HBV-related disorders.

Our study also has some deficiencies. First, we did not evaluate the specificity of serum CMTM2 for HBV infection. It would be better to compare HBV infection with other disease using the same set of analysis, such as chronic hepatitis C or nonalcoholic steatohepatitis. Moreover, the sample size included is small, which may cause selectivity bias, so the sample size needs to be expanded to further support this study.

## 5. Conclusions

In general, our findings strongly indicate that the patients with HBV-related liver diseases showed significantly decreased serum CMTM2 levels than the healthy group. And we also show that serum CMTM2 correlate with HBV DNA load and may predict the patients with HBV-related disorders from healthy individuals. In HBV-infected cell model, depressed expression of CMTM2 was attributed to that HBx-activated ubiquitin-proteasome system increase CMTM2 degradation. These results provide a novel clinical viewpoint that serum CMTM2 may be adopted as a new biomarker candidate that contributes to the initiation and progression of HBV-related disorders.

## Figures and Tables

**Table 1:** Clinical characteristics of subjects in this study.

Parameter^∗^	HC	CHB	HBLC	HCC
Number of subjects (proportion of HBV DNA assessment, %)	52	69 (84%)	77 (39%)	53 (15%)
Age (range)	21-91	21-78	30-75	35-75
Year (mean)	48.1	44.1	54.4	56
Sex (male: female)	40 : 12	53 : 16	51 : 26	48 : 5
HBsAg positive	No	Yes	Yes	Yes
CMTM2 (pg/ml)	1379.8 ± 356.5	843.2 ± 325.0	866.6 ± 420.0	831.9 ± 343.0
ALT (U/l)	17 ± 6.6	235.1 ± 324.2	42.9 ± 49.4	56.0 ± 58.9
AST (U/l)	19.75 ± 4.83	173.7 ± 206.7	60.9 ± 69.8	76.9 ± 91.1
HBV DNA (copies/ml)	N/A^†^	8480136 ± 46763772	5508195 ± 20426271	16381 ± 30431

^∗^For CMTM2 values, ALT, AST, HBV DNA titres, data are presented as mean ± SD. ^†^N/A not available. HC: healthy control; CHB: chronic hepatitis B; HBLC: hepatitis B associated liver cirrhosis; HCC: hepatocellular carcinoma; HBsAg: hepatitis B surface antigen; CMTM2: CKLF-like MARVEL transmembrane domain containing 2; ALT: alanine transaminase; AST: aspartate aminotransferase; HBV DNA: hepatitis B virus deoxyribonucleic acid.

## Data Availability

No data were used to support this study.
